# Pesticide knowledge and attitude among the potato growing farmers of Bangladesh and determinant factors

**DOI:** 10.3389/fpubh.2024.1408096

**Published:** 2024-07-31

**Authors:** Mohammad Zabed Hossain, Fahliza Ferdous, Md. Israt Rayhan

**Affiliations:** ^1^Department of Botany, University of Dhaka, Dhaka, Bangladesh; ^2^Institute of Statistical Research and Training, University of Dhaka, Dhaka, Bangladesh

**Keywords:** agrochemicals, practices, health and environmental risks, regression analysis, socio-demographic characteristics

## Abstract

The study aimed to assess the extent of pesticide use among potato-growing farmers in Bangladesh and its relationship with their knowledge, attitude, and socio-demographic characteristics. Data were collected from 553 farmers using a semi-structured questionnaire through multistage random sampling. Bivariate analysis was conducted to examine the relationship between the frequency of pesticide use and various socio-demographic factors. Results showed that out of 321 different pesticide brands reported, 50.5% were registered, while 47.7% were unregistered and 1.9% were banned. Among the registered pesticides, 5.6% were highly hazardous, 24.8% were moderately hazardous, and 6.2% were slightly hazardous as per World Health Organization category. A high percentage (96%) of farmers reported using pesticides in their fields, with 16.6% applying pesticides more than five times in a cropping season. Data revealed that majority of the farmers were aware of the negative effect of pesticides on health and environment. Most farmers used hand towels (77.9%) and ordinary shirts (70.0%) to cover their bodies to avoid pesticide exposure. Inappropriate disposal of empty pesticide containers was also observed. Negative binomial regression analysis revealed significant positive associations between the frequency of pesticide application and potato productivity, rate of fertilizer application, area of land owned by farmers, and their knowledge about the negative effects of pesticides on human health. The study suggests adopting integrated pest management practices, developing pest-resistant potato varieties, ensuring safe handling practices and disposal as well as stringent enforcement of laws to mitigate pesticide externalities and hence ensure sustainability in agriculture.

## 1 Introduction

During the Green Revolution, pesticides played a pivotal role in boosting food production by safeguarding crops against pests and diseases. However, the unintended consequences of pesticide use on human health and the environment have raised significant concerns for the sustainability of agriculture. Studies by Mack et al. (1), John and Babu (2), Taiwo (3), Hawkins et al. (4), Shammi et al. (5), Bourguet and Guillemaud (6), Malaj et al. (7), Sala and Bocchi (8), Pimentel (9), and Saeed et al. (10) have highlighted various negative impacts, including public health risks, pest resistance, biodiversity loss, yield reduction, increased defense costs, and environmental pollution. The degradation of soil quality owing to pesticides, as indicated by Dudley et al. (11), Shao and Zhang (12), and Del Prado-Lu (13) poses a significant threat to agroecosystem productivity. Moreover, economic losses due to health impairments (14) and price hikes in crop products (15, 16) further underscore the detrimental effects of pesticides. To mitigate these externalities, it is imperative to comprehend the factors influencing pesticide application in crop production and explore alternative, sustainable approaches.

Potato (*Solanum tuberosum* L.) holds a significant position as the third most produced and consumed crop globally, trailing only wheat and rice (17). However, the prevalence of pests and diseases in potato cultivation has led to the widespread use of pesticides (18). The popularity of high-yielding potato varieties is on the rise due to their potential to enhance production, livelihoods, income generation, and food security resilience at the household level (19). Nonetheless, these varieties are heavily reliant on chemical inputs (12, 20, 21). Additionally, weed infestations significantly impact potato yield and quality (22), prompting the use of herbicides alongside pesticides. Sookhtanlou et al. (23) have highlighted a concerning trend of increasing pesticide use in potato fields. The inappropriate and prolonged use of pesticides has led to the development of resistance in pests (24–26), thereby exacerbating the issue and prompting farmers to misuse and overuse these chemicals. Addressing these challenges requires a multifaceted approach that considers sustainable alternatives to chemical-intensive potato cultivation practices.

The indiscriminate and unsafe use of pesticides can be attributed to various factors, including poor knowledge, lack of education, and farmers’ behavior (27). Long-standing beliefs, perceptions, and socio-demographic characteristics also play a role in shaping pesticide practices. Studies have shown a significant association between pesticide use and farmers’ education, knowledge, and land ownership (21, 28–31). Furthermore, there is a reported correlation between pesticide knowledge and the safety measures adopted by farmers (32). However, despite having knowledge about pesticide hazards, some farmers still demonstrate poor safety measures and practices, as noted by Jallow et al. (33), indicating the complexity and confounding nature of the determinants of pesticide use. It is worth noting that factors influencing pesticide use vary widely across regions and countries and may not follow similar patterns (35). Understanding these diverse factors is crucial for developing targeted interventions to promote safe and sustainable pesticide practices among the farmers.

In Bangladesh, potato holds a significant position, contributing up to 55% of the total vegetable production, ranking second only to rice, with an increasing area of cultivated land over the years (36, 37). The yield of potato in Bangladesh has increased from 18.09 to 21.84 MT/ha over the period from 2010 to 2022 (38). However, this growth raises concerns about agricultural sustainability due to the prevalent use of pesticides. Despite this, there has been a lack of comprehensive data on the extent of pesticide use by potato farmers and the factors driving it. This study aimed to address this gap by investigating the types of pesticides used by farmers, as well as their knowledge and attitudes toward pesticide use. Additionally, the research sought to identify socio-demographic factors influencing pesticide usage during potato cultivation in Bangladesh. By shedding light on these aspects, the study aimed to provide valuable insights for developing strategies to promote safer and more sustainable agricultural practices in the potato farming sector.

## 2 Materials and methods

### 2.1 Study site description

Among the 21 most potato producing districts in Bangladesh (39), two thirds (14) were selected randomly in order to capture the heterogeneity among potato cultivating farmers, land types (e.g., flooded or non-flooded), agro-ecological zones, and geographical distribution (e.g., latitude and longitude) in the study area. The selected districts were under four administrative divisions namely Rangpur (Panchagarh, Thakurgaon, Nilphamari, Lalmonirhat, Kurigram, Dinajpur, and Rangpur), Rajshahi (Joypurhat, Naogaon, and Bogura), Dhaka (Manikganj, Munshiganj, and Shariatpur), and Barishal (Bhola). The latitude of the study area ranged from 22.1785° in Bhola to 26.0418° in Thakurgaon whereas the longitude ranged from 88.4283° in Thakurgaon to 90.7101° in Bhola. This broad geographic coverage ensured the inclusion of diverse environmental and agricultural conditions, enhancing the representativeness and reliability of the study findings.

### 2.2 Sampling design

A cross-sectional study with multistage sampling design was followed to collect data (40). The design of this study applied a quantitative method by interviewing potato growing farmers from face to face. Initially, from each of the selected districts, the five most significant potato-producing Upazilas (sub-districts) were identified. Subsequently, from each Upazila, eight farmers from eight different unions (village-level administrative units) were selected randomly from a list of potato farmers provided by the Upazila Agriculture Extension Officers. This list was prepared by the Agriculture Extension Officers by taking information on names and addresses of the farmers who used to come to them for agriculture related services at different times during the cropping season of potato. In total, 40 farmers were targeted from each district, resulting in a total sample size of 560 farmers across all 14 districts. However, due to logistical challenges arising from the onset of the COVID (Corona Virus Disease)-19 pandemic and associated lockdown measures, data collection was feasible for 553 farmers instead of the initially planned 560. Data collection was finished before starting of the countrywide lockdown due to COVID-19 pandemic. Despite this slight deviation from the intended sample size, the study aimed to maintain rigor and reliability in its findings through careful sampling and data collection procedures.

### 2.3 Data collection

The data collection process involved utilizing a semi-structured questionnaire administered through field-level interviews. The questionnaire covered various aspects, including pesticides, farmers’ socio-demographic profiles, and behavioral factors. Specifically, information on pesticide names, the frequency of spraying in a cropping season, socio-demographic profiles, handling practices, and knowledge regarding health and environmental hazards of pesticides were collected. Data collection was timed strategically, occurring 1 week before and within 1 week after harvesting to ensure farmers could recall information accurately. Additionally, to validate the data collected from farmers, visits were made to retail sellers and dealers of pesticides in the study areas. To supplement primary data, secondary sources such as online databases, published scientific papers, and documents from relevant organizations were utilized. The information gathered from these sources included generic names, chemical compositions of pesticides, and additional contextual data. To ensure data accuracy and consistency, rigorous checks were conducted through repeated field visits, especially in cases of inconsistency. The data collection process took place during the months of February and March 2020.

Regarding farmers’ attitudes and knowledge about environmental and health hazards associated with pesticides, approaches described in previous studies (33, 34) were followed. Both closed and open-ended questions were asked to the respondents for the collection data. In the closed questions, there were multiple choices to answer and farmers were asked to select either one or more appropriate answers as per their opinion or attitude on an issue. Names of the pesticides brands farmers mentioned during interview were verified by checking the list obtained from the local dealers and agriculture extension officials. For the self-reported symptoms owing to pesticide exposure, farmers were asked to mention sickness or sicknesses they felt after spray within the past year prior to the date of interview. The questionnaire was designed in consultation with relevant stakeholders, including agricultural extension officers, scientists, and researchers. It was crafted in the native language (Bengali) to facilitate clear communication between farmers and interviewers. Prior to field data collection, a week-long pilot study was conducted among 20 farmers to refine the questionnaire and survey techniques. Data obtained from the pilot study was not included in this study. Field data collectors underwent a 10-day training program to ensure uniformity and accuracy in data collection.

### 2.4 Statistical analysis

Given that the majority of respondents (96.4%) reported using pesticides, the number of non-users (control group) was minimal, comprising only 3.6% of the total sample. Consequently, utilizing a probit or logit model, which typically require a balance between the control and treatment groups, did not yield meaningful results in signifying covariates. Instead, regression analysis was conducted with the frequency of pesticide application as the dependent variable. Initially, bivariate analysis was performed to assess the relationship between the frequency of pesticide use and other covariates (independent variables). Variables showing significance in this analysis were then included in subsequent regression analyses. Given that the dependent variable represented count data, Poisson and Negative binomial regression models were considered. However, both deviance and Pearson Goodness of Fit tests indicated overdispersion in the count data. Therefore, the negative binomial regression model (41) was preferred over the Poisson regression to account for this overdispersion and provide more accurate estimates of the relationships between the frequency of pesticide use and the selected covariates.

## 3 Results

### 3.1 Socio-demographic statistics of the respondents

All respondents in the study area were male farmers with an average age of 44.5 years. The literacy levels varied, with 15.9% of respondents being illiterate (having no schooling experience), while the remaining had different levels of education: up to class five (20%), higher secondary (9%), and tertiary education (7%). Regarding the main profession, approximately 89% of respondents were engaged in agriculture, followed by 6.7% in business, and the remainder in other professions. On average, each respondent had a *per capita* area of 0.14 ha for homestead and 0.97 ha for agricultural land. These demographic and socioeconomic characteristics provide insights into the composition of the sample population and offer context for understanding their behaviors and practices related to pesticide use in potato cultivation.

### 3.2 Pesticides used in potato cultivation

In the potato fields of the study area, a total of 321 different pesticide brands were reported, with 50.2% registered, 48.0% unregistered, and 1.2% banned according to the guidelines of the government of Bangladesh. Classification of pesticides based on the chemical families depicted that carbamates comprised the majority of pesticides at 33.5%, followed by organophosphates at 22.2%, organochlorines at 4.2%, and pyrethroids at 3.0%. Other proportion (37.1%) was contributed by mixtures of these four types of pesticides.

Of the registered pesticides, fungicides constituted the majority at 50.31%, followed by insecticides at 42.9%, herbicides at 3.1%, and other types at 10.6%, including miticides and rodenticides in the studied potato fields (Table 1). Within the registered pesticide category, 5.6% were classified by the World Health Organization (WHO) as highly hazardous (Ib), 24.8% as moderately hazardous (II), 6.2% as slightly hazardous (III), and 37.3% as unlikely to present acute hazards in normal use (U) for human health. Additionally, 10.6% pesticides were not categorized into any of these hazard levels. Furthermore, a significant number of pesticides were categorized as mixtures of the unique hazard categories mentioned above. These findings provide insights into the types, hazard levels, and compositions of pesticides used in potato cultivation in the study area, offering valuable information for assessing potential risks and formulating regulatory measures.

**Table 1:** Number of registered pesticides under different hazard category [as per WHO (42)] reported from the potato growing farmers of the study area of Bangladesh.

Hazard category^*^	Fungicide	Insecticide	Herbicide	Other	Total (%)
Ib	-	9	-	-	9 (5.6)
II	1	39	-	-	40 (24.8)
III	10	-	-	-	10 (6.2)
III + II	4	1	-	-	5 (3.1)
U + III	18	-	-	-	18 (11.2)
U + II	1	1	-	-	2 (1.2)
U	46	5	4	5	60 (37.3)
Not classified	1	14	1	1	17 (10.6)
Total	81	69	5	6	161 (100.0)

^*^Ia, Extremely hazardous; Ib, Highly hazardous; II, Moderately hazardous; III, slightly hazardous; U, Unlikely to present acute hazard in normal use. “-” indicates not found.

### 3.3 Farmers’ knowledge about pesticides and associated negative effects

Farmers indicated various sources from which they gained knowledge about pesticide handling practices (Table 2), with the majority citing fellow farmers (83.2%), followed by pesticide dealers (46.3%) and agriculture staff (31.1%). Fewer respondents mentioned obtaining knowledge from instructions written on the pesticide label (23.5%), manufacturers’ representatives (2.2%), government training centers (2.0%), and NGO training centers (0.2%). Regarding the habit of reading instructions on the label of pesticide containers, the majority (80.3%) affirmed doing so, while a minority (19.7%) did not. In terms of the perceived environmental effects of pesticides, most respondents identified biodiversity loss (69.1%) as the primary concern, followed by environmental pollution (15.9%) and human health impairment (8.9%) (Table 2). Some respondents mentioned foul smell (0.2%) as an effect, while a small percentage believed there were no side effects (0.7%). Additionally, a proportion of respondents admitted to not knowing (15.9%) about the environmental effects of pesticides.

**Table 2:** Knowledge about pesticides and their negative effects on environment and human health among the potato growing farmers of the study area of Bangladesh (*N* = 553).

Question and response	*n*	%
From where do you know about the safe handling of pesticides?^*^		
Fellow farmers	460	83.2
Pesticides dealers	256	46.3
Agriculture officers	172	31.1
Instructions on the label	130	23.5
Manufacturers’ representatives	12	2.2
Government training centers	11	2.0
NGO training centers	1	0.2
*Do you read the instructions written on the containers of pesticides?*		
Yes	444	80.3
No	109	19.7
What are the effects of pesticides on environment?^*^		
Biodiversity loss	382	69.1
Environmental pollution	88	15.9
Human health impairment	49	8.9
No side effects	4	0.7
Foul smell	1	0.2
Do not know	88	15.9

^*^ indicates multiple responses.

### 3.4 Farmers’ practices about pesticide use

Of the respondents, 96.4% said that they used pesticides for potato cultivation and the frequency of spray in a cropping season varied from a single time (27.2%) to two times (27%), three times (12.4%), four times (10.1%), 5 times (6.8%), and more than 5 times (16.8%) (Table 3).

**Table 3:** Frequency of pesticide spray by the farmers in a cropping season of potato in the study area of Bangladesh (*N* = 553).

Question and response	*n*	%
*Do you use pesticides in potato cultivation?*		
Yes	533	96.4
No	20	3.6
*How many times do you apply pesticides in your potato field?*		
1 time	150	27.1
2 times	149	26.9
3 times	68	12.3
4 times	56	10.1
5 times	38	6.9
>5 times	72	13.0

As shown in Table 4, most farmers used to use a piece of cloth (locally known as *Gamcha* or *Rumal*) as mask (77.9%) to cover their mouth followed by ordinary shirts (70%) to cover their body, gloves (19.2%), additional cloths over shirts as apron (3.8%), gum boot (1.1%), and goggles (0.4%). On the other hand, only five farmers (0.9%) said that they did not use cloths (meaning upper parts of body exposed) to protect their body from exposure to pesticides. Of all the respondents, 23.1% said that they used to mix pesticides at home while the rest of them (76.9%) mentioned that they did it on-farm before spraying. In response to the question what did they do with the empty pesticide containers (e.g., bottles, packets, and bags) after they finished using those containers, most frequently (47.2%) cited answer was “Throw away” followed by “Burying under soil” (29.7%), “Burning” (24.1%), and “Sell out” (7.1%). Besides, few other responses such as “Use for domestic purposes” (1.1%), “Use for agricultural purposes” (0.2%), and “Store at home for further use” (0.2%) were also reported.

**Table 4:** Safety measures followed and disposal of empty pesticide containers by the potato growing farmers in the study area of Bangladesh (*N* = 553).

Question and response	*n*	%
What personal protective equipment (PPE) do you use while applying pesticides?^*^		
Masks (hand towels)	431	77.9
Shirts	387	70.0
Gloves	106	19.2
Apron (Additional cloths over shirts)	21	3.8
Boot	6	1.1
Nothing	5	0.9
Goggles	2	0.4
*Where do you prepare mixture of pesticides?*		
At field	425	79.7
At home	108	20.3
What do you do with the empty pesticide containers?^*^		
Throw away	261	57.0
Burying under soil	164	24.9
Burning	133	18.6
Sell out	39	5.0
Use for domestic purposes	6	0.5
Use for agricultural purposes	1	0.1
Store at home for further use	1	0.1

^*^indicates multiple responses.

### 3.5 Farmers’ self-reported toxicity symptoms and actions toward exposure

As shown in Table 5, the most frequently reported symptoms experienced by farmers after handling pesticides included headache (38.7%), nausea (34.7%), itchy eyes (25.0%), fatigue (20.4%), skin irritation (19. 9%), feeling no appetite (11.4%), and shortness of breath (5.3%). Additionally, a few farmers mentioned experiencing fever, hair fall, and stomach ache due to pesticide handling. Overall, 84.3% of the farmers reported that they experienced pesticide poisoning. When feeling sick after pesticide exposure, the majority of farmers (40.9%) reported taking rest as a form of treatment. Others mentioned taking medicine by their own prescription (15.2%), consulting with physicians (14.5%), or doing nothing (8.1%). These responses illustrate the range of symptoms experienced by farmers as well as their approaches to managing pesticide-related health issues.

**Table 5:** Self-reported toxicity symptoms and actions to pesticides exposure among the potato growing farmers in the study area of Bangladesh (*N* = 553).

Question and response	*n*	%
What sickness do you feel after handling pesticides?*		
Headache	214	38.7
Nausea	192	34.7
Itchy eyes	138	25.0
Fatigue	113	20.4
Skin irritation	110	19.9
Feeling no appetite	63	11.4
Shortness of breath	32	5.8
Fever	2	0.4
Stomach ache	1	0.2
Hair fall	1	0.2
No sickness	87	15.7
*What treatment do you take if you feel sick after using pesticides?*		
Take rest	226	40.9
Take medicine on own prescription	84	15.2
Consult with physicians	82	14.8
Do nothing	45	8.1

^*^ indicates multiple responses.

### 3.6 Determinants of pesticide application by the potato farmers

Results showed that the negative binomial regression analysis yielded better fit (Table 6) compared to Ordinary Least Square (OLS) (Supplementary Table 1) and Poisson analysis (Supplementary Table 2) as evidenced by lower AIC and BIC values. The results indicated positive associations between the frequency of pesticide application and variables such as investment of money for potato cultivation, area of land owned by farmers, and level of secondary education (SSC). Results showed that one unit increase in the amount of money spent for cultivation of potato was associated with approximately a 10% increase in the frequency of pesticide use. Similarly, one unit increase in area of land owned by a farmer and level of secondary education (SSC) was linked to an increase of 10 and 13%, respectively increase in the frequency of pesticide sprays. Although level of secondary education showed significant correlation with frequency of pesticide application other levels of education such as below class 5, up to class 5, up to class 8, Bachelor degree, and Master degree did not show such correlation. Additionally, despite farmers’ awareness of the negative effects of pesticides on human health, they continued to use them in the field.

**Table 6:** Negative binomial regression analysis on the frequency of pesticide application by the potato growing farmers over the covariates in the study area of Bangladesh.

Times pesticides applied (Dep.)	IRR	Standard error	*Z*	*P* > |*z*|	[95% confidence interval]	
Money spent	1.000	0.000	2.02	0.043	1.000	1.000
Age of farmer	0.999	0.002	−0.22	0.825	0.995	1.004
Area of land	1.000	0.000	2.27	0.023	1.000	1.000
Education (Ref: no education)						
Below class 5	1.094	0.097	1.01	0.312	0.919	1.301
Up to class 5	1.125	0.090	1.47	0.142	0.962	1.315
Up to class 8	1.129	0.092	1.50	0.135	0.963	1.325
SSC	1.264	0.119	2.48	0.013	1.051	1.520
HSC	1.107	0.121	0.93	0.354	0.893	1.372
Bachelor	1.185	0.138	1.46	0.144	0.944	1.488
Master	1.396	0.253	1.84	0.066	0.979	1.990
Know impact of pesticide on health	1.272	0.069	4.43	0.000	1.144	1.415
Know impact of pesticide on biodiversity	1.023	0.066	0.35	0.724	0.901	1.162
AIC	3790.65					
BIC	3851.04					

Dep., Dependent variable; IRR, Incidental rate ratio; SSC, Secondary School Certificate; HSC, Higher Secondary Certificate; AIC, Akaike’s information criteria; BIC, Bayesian information criteria.

An alarming finding from the analysis is that even among farmers who were aware of the negative impacts of pesticides on health, there was a 1.3 times higher incidence rate of pesticide use compared to those who were not aware of negative impacts. Overall, these findings highlight the complex relationship between various factors influencing pesticide use in potato cultivation, including land ownership, investment, and farmers’ awareness of pesticide hazards.

## 4 Discussion

The findings of the present study underscore the alarming extent of pesticide use in potato cultivation of Bangladesh. Over 96% of potato farmers reported applying pesticides, using a staggering total of 321 different pesticide brands, including both registered and unregistered, and even banned ones. Of particular concern is the lack of information regarding the chemical composition and hazard category of these unregistered chemicals. The use of unregistered pesticides poses significant risks, potentially exacerbating environmental problems through overuse and the inclusion of banned chemicals in agricultural fields. It is troubling to note that more than 36% of the registered pesticides used were classified as health hazardous according to WHO (42) classification. Furthermore, approximately 60% of these pesticides were carbamates, organophosphates, and organochlorines—persistent and bioaccumulating substances known to impact the reproductive and developmental stages of humans and animals (43–46). The results that 84.3% of the respondents experienced poisoning while spraying pesticides demonstrated an alarming scenario for the health of the potato growing farmers of the study area. Another worrisome finding is the frequency of pesticide application, with farmers spraying pesticides multiple times throughout the cropping season. Although we did not ask the respondents whether the same persons did this job of spraying pesticides year after year, we could assume that the same group of people did this job in a specific area. Because, this kind of job requires special knowledge on how to operate spray machine and prepare pesticide solutions. Further, it also needs physical strength enough to carry spray machine and bear the nuisance of noise and exposure to hazards while operating machine. A long-term exposure to hazardous pesticides is likely to impact farmers’ health negatively. Thus, indiscriminate use of pesticides poses a threat to human health, the natural environment, and the overall productivity of agroecosystems in Bangladesh (47). These results underscore the urgent need for effective regulation, monitoring, and enforcement of pesticide use, as well as increased awareness and adoption of sustainable agricultural practices that minimize reliance on harmful chemicals. Addressing these issues is essential to safeguarding human health, preserving environmental integrity, and ensuring the long-term sustainability of agricultural systems in Bangladesh.

The prevalent use of unsafe pesticides in potato cultivation in Bangladesh can be attributed to several factors. Firstly, the affordability and easy accessibility of these pesticides make them an attractive option for farmers. Additionally, farmers may prefer pesticides with fast-acting pest control properties, as these chemicals often exhibit higher lethality against pests. However, the short-term benefits of using such pesticides may come at a significant long-term cost. Overuse of pesticides can lead to the development of resistance in crops against these chemicals (4), rendering them ineffective over time. This phenomenon underscores the importance of adopting sustainable pest management practices to prevent the escalation of resistance issues. Despite the existence of the Pesticides Act, 2018 (Law No. 24 of 2018), which addresses various aspects of pesticide regulation, including registration, import policies, and guidelines for storage and use, pesticide use remains widespread in potato cultivation in Bangladesh. Given these concerns, it is imperative for the relevant authorities within the Government of Bangladesh to take proactive measures to regulate and control the sale and usage of unregistered and hazardous pesticides. This includes stringent monitoring mechanisms to ensure compliance with safety and efficacy standards. Furthermore, there is a need to reassess the approval status of pesticides currently in use to determine their effectiveness in pest control while minimizing adverse impacts on human health and the environment. By implementing these measures, the Government can safeguard the interests of both farmers and consumers while promoting sustainable agricultural practices in the country.

The study revealed a concerning gap between farmers’ knowledge of the negative effects of pesticides on the environment and their actual practices regarding safe handling and disposal. Despite being aware of these risks, farmers’ attitudes toward proper pesticide handling and disposal were unsatisfactory. This discrepancy highlights the need for formal and institutional mechanisms to provide farmers with pesticide knowledge and hands-on training on safe handling and disposal practices. In the absence of such mechanisms, inappropriate handling and disposal of pesticides and empty containers have created opportunities for exposure and the spread of residues into the surrounding environment. The reuse of empty pesticide containers further exacerbates these risks, posing potential health hazards to individuals. Unfortunately, inappropriate handling of empty containers is not uncommon in developing countries, as evidenced by other studies (18, 48–50). Therefore, it is essential that the Government of Bangladesh implements interventions aimed at raising mass awareness, motivating farmers, and providing hands-on training on pesticide knowledge and handling practices. This approach not only protects human health and the environment but also promotes sustainable agricultural development in the long run.

The self-reported symptoms attributed to pesticide exposure underscore the vulnerability of farmers’ health to the harmful effects of pesticides, both in the short and long term. While this study did not examine the prevalence of chronic effects such as cancer and asthma resulting from pesticide exposure, it is likely that such impacts exist due to prolonged exposure over time (35, 46).

The result found in the present study that all respondents were male farmers in the survey area was not in alignment with the current situation of the contribution of women in agricultural workforce of Bangladesh. Because, about 60% of the total workforce in agriculture is female and the proportion of their contribution in this sector is increasing over the years compared to that of male (51). The gap between the proportions of male farmers as respondents and the average contribution of female in agricultural activities of the country might be attributed to a number of factors including under-recognition of the women’s role in this sector. Male farmers still dominate in decision making in agricultural sector and their names are recognized as main farmers and therefore are listed as farmers in the local Agriculture Extension Offices. Another factor behind this gap might be the reason of higher financial investment for a number of purposes including quality seeds, fertilizers, pesticides, irrigation and cold storage facility for preservation of potato (52). Usually, women has less decision making power in the current social structure of Bangladesh (53). Moreover, involvement of women in agriculture was found to be inversely related with their educational background in Bangladesh (51). For this reasons, although female farmers were engaged in both pre- and post-harvest activities and its preservation as well, they were not decision makers in potato cultivation. Additionally, since the application of pesticide is very common in potato cultivation and the job of spraying pesticides in the field is laborious requiring special physical strength and precautions such activities are usually performed by the male farmers. Therefore, it was likely that all respondents of the present study were male farmers.

The lack of appropriate personal protective equipments (PPEs) among farmers further exacerbates their vulnerability to pesticide poisoning. Pesticides can cause dermal and respiratory exposure, leading to a range of health issues (46). The absence of adequate protective measures may stem from the unavailability or unaffordability of PPEs for farmers. During field visits and interactions with pesticide dealers, no evidence of PPEs being readily available for sale to users was observed. Therefore, it is crucial to make PPEs more accessible and affordable for farmers. By ensuring the availability and affordability of PPEs, policymakers and stakeholders can help mitigate the risks associated with pesticide exposure and safeguard the health and well-being of agricultural workers (54). Additionally, comprehensive education and training programs on the proper use of PPEs should be implemented by the Government to promote their effective utilization among farmers. These efforts are essential for reducing pesticide-related health hazards and fostering a safer working environment in agriculture.

The findings from the binomial regression analysis suggest that farmers with higher levels of education, land area and financial ability tend to apply more pesticides in their potato fields. These results aligned with previous studies, which also reported that use of pesticides in the crops increased with the increase of education level (21) and land area owned by the farmers (55). From these results, it appears that farmers with greater financial resources may be willing to invest more money in pesticide application in the hope of achieving higher economic returns. However, these results indicate that simply having education and knowledge about pesticide externalities is not sufficient to control pesticide use effectively. It is crucial for farmers to be self-motivated to adopt sustainable agricultural practices, and regulatory bodies must ensure monitoring and strict enforcement of laws to minimize pesticide hazards. It is also essential to educate farmers about the long-term effects of pesticides, highlighting the trade-offs between short-term economic gains and potential health and environmental risks.

## 5 Conclusion

The scenario of pesticide use among potato growing farmers of Bangladesh is alarming due to the widespread use of unregistered and health hazardous pesticides. Results also revealed inappropriate handling and disposal practices of pesticides by farmers that created risks for human health and surrounding environment. The study further demonstrated that despite their knowledge about the negative externalities associated with pesticides, farmers continued to apply hazardous chemicals to protect their crops against pests and diseases to enhance production. A comprehensive approach including development of pest-resistant potato varieties, biocontrol of pests, subsidies to the farmers not to use pesticides, motivation and promotion to both the supply and demand sides, stringent regulation of pesticide production, and marketing and application in the field were suggested for sustainable potato cultivation.

## Data availability statement

The raw data supporting the conclusions of this article will be made available by the authors, without undue reservation.

## Ethics statement

Ethical review and approval was not required for the study on human participants in accordance with the local legislation and institutional requirements. Written informed consent from the patients/participants or the patients'/participants' legal guardian/next of kin was not required to participate in this study in accordance with the national legislation and the institutional requirements.

## Author contributions

MH: Conceptualization, Data curation, Funding acquisition, Investigation, Methodology, Project administration, Resources, Supervision, Validation, Visualization, Writing – original draft, Writing – review & editing. FF: Investigation, Project administration, Writing – original draft. MR: Data curation, Formal analysis, Methodology, Software, Validation, Writing – review & editing.

## Supplementary material

SUPPLEMENTARY TABLE 1 Ordinary least square (OLS) regression analysis on the frequency of pesticide application by the potato growing farmers in the study area of Bangladesh.

SUPPLEMENTARY TABLE 2 Poisson regression analysis on the frequency of pesticide application by the potato growing farmers in the study area of Bangladesh.
