# Supplementary material for: Pesticide knowledge and attitude among the potato growing farmers of Bangladesh and determinant factors
Source: Front Public Health. 2024 Jul 31;12:1408096. doi: 10.3389/fpubh.2024.1408096 (PMC11322349; doi:10.3389/fpubh.2024.1408096)
Supplement: SUPPLEMENTARY TABLE 1 — Ordinary least square (OLS) regression analysis on the frequency of pesticide application by the potato growing farmers in the study area of Bangladesh. [file Table_1.docx]

**supplementary Table 1**

| Times pesticide applied (Dep.) | Coefficient | Standard error | t | P>\|t\| | [95% confidence  interval] | |
| --- | --- | --- | --- | --- | --- | --- |
| Money spent | 0.002 | 0.001 | 1.97 | 0.049 | 5.060 | 0.003 |
| Age of farmer | 0-.007 | 0.041 | -0.16 | 0.871 | -0.086 | 0.073 |
| Area of land | 0.001 | 0.001 | 1.90 | 0.058 | -0.000 | 0.003 |
| Education  (Ref: no education) |  |  |  |  |  |  |
| below class 5 | 1.028 | 1.559 | 0.66 | 0.510 | -2.034 | 4.090 |
| up to class 5 | 1.281 | 1.404 | 0.91 | 0.362 | -1.478 | 4.039 |
| up to class 8 | 1.330 | 1.432 | 0.93 | 0.353 | -1.482 | 4.143 |
| SSC | 3.608 | 1.664 | 2.17 | 0.031 | .3310 | 6.876 |
| HSC | 1.327 | 1.937 | 0.69 | 0.494 | -2.478 | 5.132 |
| Bachelor | 2.045 | 2.059 | 0.99 | 0.321 | -2.000 | 6.090 |
| Master | 4.663 | 3.237 | 1.44 | 0.150 | -1.696 | 11.022 |
| Know impact of  pesticide on health | 3.290 | 0.958 | 3.43 | 0.001 | 1.408 | 5.172 |
| Know impact of  pesticide on biodiversity | 0.221 | 1.138 | 0.19 | 0.846 | -2.016 | 2.457 |
| AIC | 4157.51 |  |  |  |  |  |
| BIC | 4213.59 |  |  |  |  |  |

Dep.: dependent variable, SSC: Secondary School Certificate, HSC: Higher Secondary Certificate, AIC: Akaike’s information criteria, BIC: Bayesian information criteria
