# Supplementary material for: Pesticide knowledge and attitude among the potato growing farmers of Bangladesh and determinant factors
Source: Front Public Health. 2024 Jul 31;12:1408096. doi: 10.3389/fpubh.2024.1408096 (PMC11322349; doi:10.3389/fpubh.2024.1408096)
Supplement: SUPPLEMENTARY TABLE 2 — Poisson regression analysis on the frequency of pesticide application by the potato growing farmers in the study area of Bangladesh. [file Table_2.docx]

**supplementary Table 2**

| Times pesticide applied (Dep.) | IRR | Standard error | z | P>\|z\| | [95% confidence  interval] | |
| --- | --- | --- | --- | --- | --- | --- |
| Money spent | 1.000 | 0.000 | 5.10 | 0.000 | 1.000 | 1.000 |
| Age of farmer | 1.000 | 0.001 | -0.32 | 0.750 | 0.998 | 1.002 |
| Area of land | 1.000 | 0.000 | 5.08 | 0.000 | 1.000 | 1.000 |
|  |  |  |  |  |  |  |
| Education  (Ref: no education) |  |  |  |  |  |  |
| below class 5 | 1.085 | 0.045 | 1.97 | 0.049 | 1.001 | 1.178 |
| up to class 5 | 1.105 | 0.041 | 2.68 | 0.007 | 1.027 | 1.189 |
| up to class 8 | 1.107 | 0.042 | 2.67 | 0.008 | 1.027 | 1.194 |
| SSC | 1.284 | 0.054 | 5.96 | 0.000 | 1.183 | 1.394 |
| HSC | 1.105 | 0.056 | 1.98 | 0.048 | 1.001 | 1.220 |
| Bachelor | 1.166 | 0.062 | 2.89 | 0.004 | 1.051 | 1.294 |
| Master | 1.378 | 0.106 | 4.15 | 0.000 | 1.185 | 1.604 |
|  |  |  |  |  |  |  |
| Know impact of  pesticide on health | 1.269 | 0.032 | 9.36 | 0.000 | 1.207 | 1.334 |
| Know impact of  pesticide on biodiversity | 1.024 | 0.031 | 0.77 | 0.439 | 0.964 | 1.087 |
| AIC | 5278.23 |  |  |  |  |  |
| BIC | 5334.30 |  |  |  |  |  |

Dep.: dependent variable, IRR: Incidental Rate Ratio, SSC: Secondary School Certificate, HSC: Higher Secondary Certificate, AIC: Akaike’s information criteria, BIC: Bayesian information criteria
